# Perceived stress and pain during breast mammography

**DOI:** 10.1192/j.eurpsy.2026.11549

**Published:** 2026-07-31

**Authors:** I. Chaari, A. Samet, A. Wissal, Y. Fourti, F. Charfeddine, L. Aribi, N. Messedi, J. Aloulou

**Affiliations:** Psychiatric department “B”, Hedi Chaker University Hospital, Sfax, Tunisia

## Abstract

**Introduction:**

Mammography is a key breast cancer screening tool. Pain during the procedure is frequent and may reduce adherence. Psychological stress could increase pain perception

**Objectives:**

To assess the relationship between perceived stress and pain during mammography.

**Methods:**

We conducted an online survey targeting the general female population in Tunisia via a sponsored Facebook campaign (December 2023–February 2024). They completed the Perceived Stress Scale (PSS-10) and rated pain (1–10) during their most recent mammogram. Correlation and regression analyses were performed.

**Results:**

A total of 278 women participated. The mean age of participants was 46 years (SD 8.9, range 20–84). The mean pain score was 5/10 (SD 2.8), with 34% of women reported severe pain (≥7/10). Higher PSS-10 scores correlated with greater pain (r = 0.34, p = 0.02). In regression, stress remained independently associated with pain (β = 0.28, p = 0.03), after adjustment for socio-demographic factors.

**Conclusions:**

Perceived stress independently predicts higher pain during mammography. Integrating stress-management interventions into breast cancer screening programs may improve procedural tolerance and adherence.

**Disclosure of Interest:**

None Declared

